# Fic and non-Fic AMPylases: protein AMPylation in metazoans

**DOI:** 10.1098/rsob.210009

**Published:** 2021-05-05

**Authors:** Bhaskar K. Chatterjee, Matthias C. Truttmann

**Affiliations:** ^1^ Cellular and Molecular Biology Program, University of Michigan, Ann Arbor, MI 48109, USA; ^2^ Department of Molecular and Integrative Physiology, University of Michigan, Ann Arbor, MI 48109, USA; ^3^ Geriatrics Center, University of Michigan, Ann Arbor, MI 48109, USA

**Keywords:** post-translational modification, filamentation induced by cAMP, non-Fic, ampylases, chaperone and neurodegeneration

## Abstract

Protein AMPylation refers to the covalent attachment of an AMP moiety to the amino acid side chains of target proteins using ATP as nucleotide donor. This process is catalysed by dedicated AMP transferases, called AMPylases. Since this initial discovery, several research groups have identified AMPylation as a critical post-translational modification relevant to normal and pathological cell signalling in both bacteria and metazoans. Bacterial AMPylases are abundant enzymes that either regulate the function of endogenous bacterial proteins or are translocated into host cells to hijack host cell signalling processes. By contrast, only two classes of metazoan AMPylases have been identified so far: enzymes containing a conserved filamentation induced by cAMP (Fic) domain (Fic AMPylases), which primarily modify the ER-resident chaperone BiP, and SelO, a mitochondrial AMPylase involved in redox signalling. In this review, we compare and contrast bacterial and metazoan Fic and non-Fic AMPylases, and summarize recent technological and conceptual developments in the emerging field of AMPylation.

## Graphical abstract

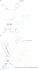

## 1. Introduction

Post-translational modifications (PTMs) may regulate proteins by activating or repressing protein function, allowing protein oligo- or monomerization or installing binding sites for allosteric interactors. The nature and impact of PTMs is diverse: some PTMs are irreversible and stable while others are transient and can be removed by dedicated enzymes in response to cellular cues. While some PTMs—such as glycosylation, lipidation or phosphorylation—have been studied extensively, others, like UMPylation, glutaminylation or AMPylation, have only recently begun to garner interest. AMPylation, also referred to as adenylylation, is conferred by AMPylases, which belong to four distinct protein families: fic domain-containing (FicD) enzymes, selenoproteins (SelO), glutamine synthetase adenylyl transferases (GS-ATase) and DrrA. These enzymes catalyse the formation of a covalent bond between the phosphate group of AMP and an accessible Ser, Thr or Tyr hydroxyl group of the target protein.

The FicD family of AMPylases is the most abundant, with more than 63 000 members (InterPro domain IPR003812 [1]) encompassing all three kingdoms of life (Archaea, Bacteria and Eukaryota). Almost all AMPylases contain the conserved Fic motif (HxFx(D/E)GN(G/K)RxxR) within their active site, which is essential for nucleotide transfer. While most FicD enzymes catalyse AMPylation, others have acquired their own novel functions. For example, the bacteriophage-encoded FicD protein, Doc, abrogates bacterial cell division and propagation through phosphorylation of the bacterial translation elongation factor EF-Tu [2,3]. Similarly, AnkX, a FicD enzyme encoded by *Legionella pneumophila*, engages in phosphocholination of Rab GTPases, disrupting endosome trafficking and intracellular secretory pathways [4]. Furthermore, some metazoan [5,6] and bacterial AMPylases [7] can also accept and transfer other nucleotides, leading to protein CMPylation, GMPylation and UMPylation.

**Figure 1.**
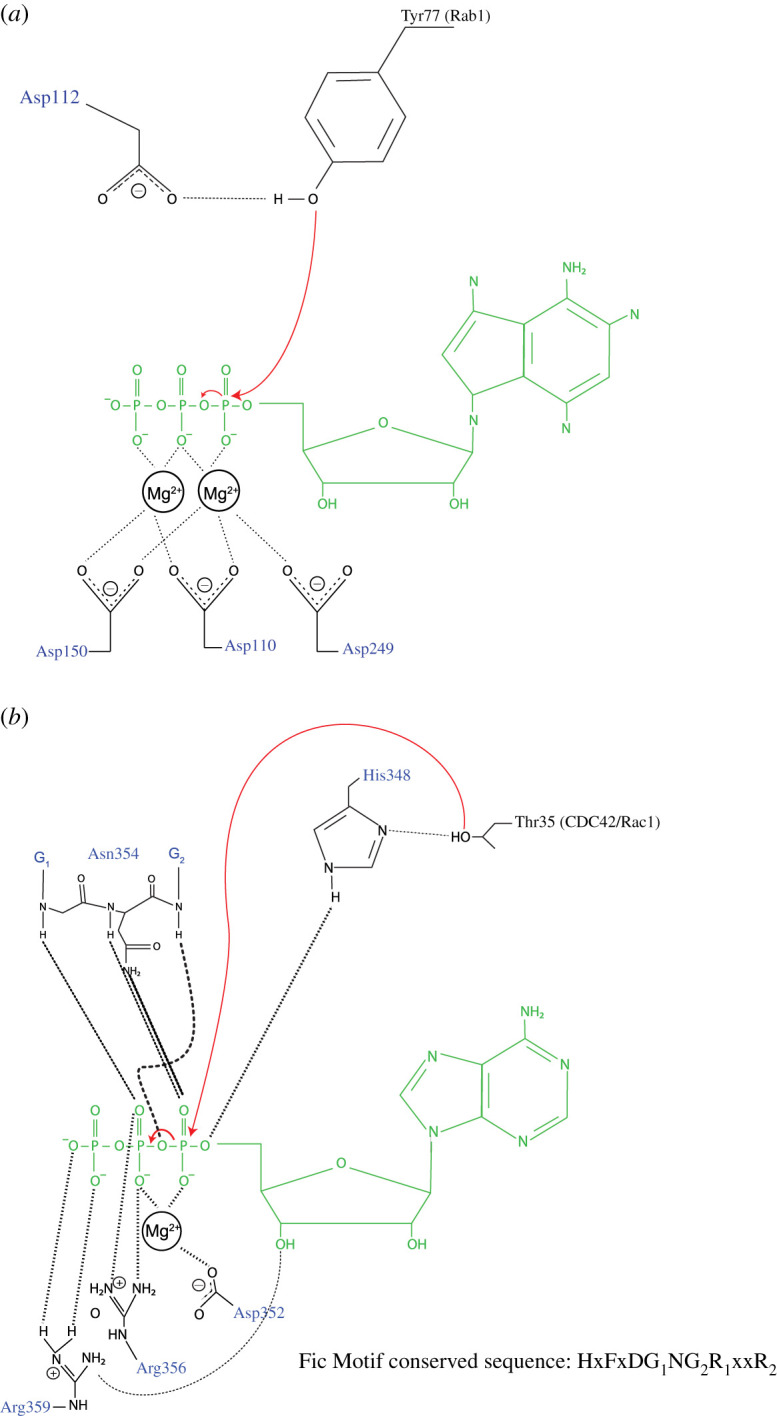
Mechanisms of target AMPylation. (*a*) Reaction scheme of Rab1 AMPylation by non-Fic AMPylase DrrA. Asp150, Asp110 and Asp249 are involved in coordination of the divalent cation; Asp112 is involved in deprotonation of the incoming target side chain (Tyr77 of Rab1). (*b*) Reaction scheme of CDC42/Rac1 AMPylation by Fic domain containing bacterial AMPylase VopS. The conserved His acts as a proton sink and deprotonates Thr35 of Cdc42/Rac1. The figure emphasizes the significance of Fic motif in coordinating the phosphates of the ATP molecule and catalysing AMP transfer. ATP molecule is depicted in green, and red arrows depict the reaction steps during AMPylation. This figure has been modified from Hedberg C. and Itzen A [8] and Gavriljuk *et.al.* [9].

In contrast to FicD enzymes, GS-ATase, DrrA and the eukaryotic pseudokinase, SelO, do not contain the conserved FicD, and employ a catalytic mechanism distinct from FicD AMPylases (figure 1). These AMPylases, together with putative additional AMPylases and deAMPylases, define the protein AMPylome, or the universe of AMPylated proteins.

In this review, we discuss bacterial AMPylases that AMPylate eukaryotic proteins as a means to subvert host cell signalling, and metazoan AMPylases that modify endogenous substrates involved in regulating protein homeostasis and other physiological processes. We will not discuss bacterial AMPylases that modify endogenous substrates which are discussed in excellent reviews by Woolery *et al*. [10] and Casey *et al.* [11]

## 2. Bacterial AMPylases that AMPylate eukaryotic targets

### 2.1. Non-FIC AMPylases

***DrrA (SidM)***, one of many Type IV secretion system effector proteins employed by the intracellular pathogen *Legionella pneumophilia,* facilitates the active recruitment of vesicles exiting the ER, which adversely affects ER-Golgi transport [12]. The function of these effector proteins is to prevent fusion of the Legionella-containing vacuole (LCV) with lysosomes, and engage in vacuole remodelling by recruiting ER-derived vesicles [12]. DrrA acts as a guanine nucleotide-exchange factor [13] and is responsible for the recruitment of a host GTPase Rab1 to the LCVs. DrrA AMPylates Rab1 on Tyr77, a conserved Tyr located in the switch II domain of the GTPase [14]. Tyr77 AMPylation prevents GTPase activating proteins (GAPs) from binding to Rab1 and promoting GTP hydrolysis [15], thus maintaining a pool of GTP-bound Rab1 on the surface of the LCVs. The C-terminal domain of DrrA contains a phosphatidylinositol-4-phosphate binding domain responsible for its attachment to the external membrane of the vacuole [16]. The N-terminal region of DrrA, which is cytotoxic when expressed alone, is structurally similar to the C-terminal adenylyl transferase domain of GS-ATase and AMPylates Rab1 *in vitro* [14]. Both GS-ATase (box 1) and DrrA belongs to the DNA-β-polymerase like family of nucelotidyl transferases that are structurally characterized by a three-stranded β-sheet involved in Mg^2+^ ion coordination and phosphate stabilization [17–19]. They also contain the degenerate motif [Gly-X_11_-**Asp/Glu**-X-**Asp/Glu**] in which the Asp residues of one such motif coordinates two Mg^2+^ ions along with another Asp or Glu residue from the following β-sheet. Asp112 of the DrrA catalytic motif [Gly-X_11_-**Asp110**-X-**Asp112**] acts as base, deprotonating the hydroxyl group of Tyr77 in Rab1, thereby enabling it to launch a nucleophilic attack on the α-phosphate of ATP, while Asp112, along with Asp150 and Asp249 (from separate β-strands), coordinate and stabilize the interaction of Mg^2+^ with the phosphates [9]. Both **Asp**110/**Asp**112 are critical for enzyme function as mutating either or both residues results in a loss of target AMPylation (figure 1*a*) [14].

Box 1. **GS-ATase: a bifunctional AMPylase.**

One of the earliest pieces of evidence of AMPylation as a PTM came from a study conducted by Anderson *et al.* who identified the bacterial enzyme glutamine synthetase adenylyl-transferase (GS-ATase) to be acting as an AMPylase [20]. GS-ATase catalyses the synthesis of L-Gln from Glu and ammonia, and is a key regulator of nitrogen metabolism [21]. GS-ATase can act bifunctionally to catalyse both AMPylation and deAMPylation of GS through two distinct catalytic sites: a C-terminal domain (residues 425–945) that catalyses AMPylation and an N-terminal domain (residues 1–423) that catalyses deAMPylation [22]. The switch between AMPylation and deAMPylation states is mediated by a signal transducing protein trimer, PII, that acts as a nitrogen sensor in bacterial cells [23,24]. When nitrogen levels are high, GS-ATase forms a complex with PII to AMPylate GS at Tyr397, thereby inhibiting its function and reducing production of L-Glutamine. However, under conditions of nitrogen starvation, PII is UMPylated by the enzyme uridyl transferase (UTase). Subsequently, the PII_UMP_-GS-ATase complex deAMPylates and thus activates GS [20,21,24–27]. Human GS is present throughout the brain and plays a major role in detoxifying astrocytes by converting excess ammonia to glutamine, and in regulating the metabolism of the neurotransmitter glutamate [28]. It is not yet known if eukaryotic GS is AMPylated and/or de-AMPylated, or what effect these modification(s) may have on astrocyte function.

Unlike effectors that AMPylate host cell targets through a conserved mechanism of catalysis mediated by the Fic domain (described in subsequent sections), GS-ATase and DrrA catalyse AMPylation through a very different catalytic fold, indicative of a divergent evolution of these two AMPylases [29].

### 2.2. Fic AMPylases

#### 2.2.1. VopS

Several years after the discovery of GS-ATase, there was a renewed interest in the function of AMPylases when VopS, a type III secretion system (T3SS) effector of *Vibrio parahaemolyticus* was found to AMPylate Rho family GTPases members Rac, Rho and CDC42 upon translocation into host cells [30]. Ectopic expression of VopS, but not of the AMPylation-deficient mutant, VopS^H348A^, induced a severely rounded phenotype eventually leading to cell death in HeLa cells. VopS-mediated AMPylation of CDC42 and Rac1 is proposed to follow a sequential reaction mechanism involving the formation of a ternary complex composed of VopS, ATP and CDC42/Rac1. In this process, VopS first transfers AMP to the conserved His348 residue, and, in a subsequent reaction, this AMP is attached to CDC42/Rac1 on Thr35 (target modification) [31]. His348 of the VopS Fic motif acts as a base in the catalytic reaction and extracts a proton from the hydroxyl group of Thr35, thereby enabling it to perform a nucleophilic attack on the α-phosphate of ATP (figure 1*b*). Asn354 and Arg356, residues embedded in the conserved Fic motif, stabilize the β- and γ- phosphate, respectively, while Asp352 coordinates the position of the Mg^2+^ ion and stabilizes it via interactions with the β- and γ-phosphates [31]. Thr35 of Rac1/CDC42 is a conserved residue located in the switch I region and plays a key role in GTP and effector binding [30]. Mechanistically, Rho GTPase AMPylation on Thr35 influences downstream signalling of NF*Κ*B and MAPK pathways, induces activation of the phagocytic NADPH oxidase complex, interferes with host cell autophagy, affects GTPase E3 ubiquitin ligase interactions required for Rac and Rho degradation and turnover, and bypasses the innate immunity arm of the host cell by suppressing the activation of pyrin inflammasomes [32–34].

#### 2.2.2. IbpA

The bacterial fibrillary surface antigen IbpA of *Histophilus somnii* is another well studied bacterial AMPylase that inhibits host cell GTPase activity. This protein contains two Fic domains (Fic1, Fic2), an arm domain important for substrate recognition [35], and a C-terminal YopT domain [36]. AMPylation of several Rho family GTPases by IbpA (table 1) results in cell death, presumably due to collapse of the cytoskeletal architecture, as observed with VopS [30,32–34]. AMPylation occurs at the conserved tyrosine residues in the switch I region of the GTPases (Tyr32 in CDC42/Rac1; Tyr34 in RhoA). Mutation of the conserved histidine residue (H3717A) in the IbpA Fic motifs did not result in cytotoxicity, confirming once again the role of this residue in AMPylation [36]. IbpA-Fic2 AMPylates GDP and RhoGDI (guanosine nucleotide dissociation inhibitor) bound inactive forms of the Rho GTPases, as well as the GTP bound active form and consequently, Rho GTPase-mediated downstream signalling pathways are inhibited [7].

**Table 1.** List of Fic domain containing proteins with known crystal structures. Modified from Truttmann & Ploegh [37] and Veyron *et al.* [38].

name	organism name	references	function/targets	structure (PDB ID)
IbpA	*Histophilus somnii*	[30,32,33]	AMPylation of Rho GTPases (Rac1, CDC42, RhoA-C, RhoG, TC10); cytotoxicity mediated by disruption of cytoskeletal regulation, repression of immune signalling pathways downstream of Rho GTPases	3N3U and 4ITR
VopS	*Vibrio parahaemolyticus*	[30,32,34]	AMPylation of Rho GTPases (CDC42, Rac1, RhoA, RhoG, TC10); cytotoxicity mediated by disruption of cytoskeletal regulation	3LET
AnkX	*Legionella pneumophila*	[4,39]	phospocholination of Rab1 and Rab35 GTPases; disrupts host cell endocyte recycling	4BEP, 4BER, 4BES and 4BET
DrrA^b^	*Legionella pneumophila*	[12,15]	AMPylation of GTPase Rab1b [14]; disrupts host intracellular vesicle transport and evades capture by lysosomes	3NKU, 3N6O, 3JZ9, 3JZA, 2WWX, 3L0I and 5O74
VbhT	*Bartonella schenbuchensis*	[40]	AMPylation of bacterial target of approximately 80 kDa; host target(s) unknown	3ZCB, 3ZC7 and 3SHG
GS-ATase^a,b^	*E. coli*	[41]	bifunctional enzyme; AMPylates and de-AMPylates bacterial glutamine synthetase through two distinct active sites	not available
EcFicT	*Escherichia coli*	[42]	host target(s) unknown; AMPylates bacterial type IIA topoisomerases and DNA gyrase; host target(s) unknown	5JFF and 5JFZ
YeFicT	*Yersinia enterocolitica*	[42]	AMPylates bacterial type IIA topoisomerases and DNA gyrase	not available
Bep1, Bep2	*Bartonella rochalimae*	[43–45]	Bep1 AMPylates Rac1/2/3 and RhoG, Bep2 AMPylates Vimentin; physiological function unknown	5EU0
NmFIC	*Neisseria meningitidis*	[46]	AMPylates endogenous DNA gyrase B; host targets unknown	2G03, 3S6A, 3SE5, 3SN9, 3ZLM,5CG7, 5CKL and 5CMT
HpFIC	*Helicobacter pylori*		physiological function unknown	2F6S
EfFIC	*Enterococcus faecalis*	[47]	host/endogenous targets unknown; possesses both AMPylation and de- AMPylation functions regulated by Ca2+ and Mg2+	5NUW, 5NV5, 6EP0, 6EP5, 6EP2, 5NWF and 5NVQ
BtFIC	*Bacteroides thetaiotaomicron*		physiological function unknown	3CUC
SoFIC	*Shewanella oneidensis*		physiological function unknown	3EQX, 3ZCN and 3ZEC
CdFIC	*Clostridium difficile*	[48]	unknown physiological targets; auto-AMPylates even in presence of inhibitory glutamate of the auto- inhibition motif	4X2E, 4X2C and 4X2D
HYPE	*Homo sapiens*	[49–53]	HYPEE234GAMPylates BiP, HSP70,HSP40, α-synuclein, eEF1A, E1F2AK2, H2-H4, ATP5A1, ATP5B, UBAP2 L, TUBB, CTSB,CTSC, CTSZ, ACP2, PNPLA3, ABHD6, TPP1, CAPZB and NSFL1C; involved in UPR activation in the ER, neuronal biogenesis, chaperone function modulation, altered aggregation of α-synuclein, ATP synthesis, cytoskeletal development and regulating protein translation	4U04, 4U0S, 4U07, 4U0U and 4U0Z
SelO^b^	*Pseudomonas syringae*	[54]	human orthologue regulates mitochondrial redox homeostasis through AMPylation of grxA and sucA	6EAC
dFIC^a^	*Drosophila melanogaster*	[55–57]	AMPylates BiP; regulates stress response in ER, required for glia- specific histamine metabolism, neurotransmitter recycling, vision and maintenance of microvilli	crystal structure not available
FIC-1	*Caenorhabditis elegans*	[6,58]	AMPylates HSP1, HSP3 and eEF1- A2; involved in regulating sensitivity to pathogenic bacteria and modulating chaperone function in cytosol and ER	5JJ6 and 5JJ7
BeP	*Bartonella quintana JK-31*		physiological function unknown	4LU4
BepC	*Bartonella henselae*	[59]	triggers actin stress fibre formation in HeLa cells	4WGJ
BepA	*Bartonella henselae*	[60]	AMPylation of eukaryotic targets of approximately 40 kDa and 50 kDa; precise function unknown	5NH2, 2VZA, 2VY3 and 2JK8
Bep5	*Bartonella claridgeiae*		physiological function unknown	4XI8
Bep8	*Bartonella sp.1–1c*		physiological function unknown	4PY3
Dde2494	*Desulfovibrio alaskensis*		physiological function unknown	4RGL
AvrB	*Pseudomonas synrigae*	[61]	affects plant immunity by targeting RIN4 [62]; AMPylation activity unknown but can potentially act as an AMPylase	1NH1, 2NUD and 2NUN
PfhB2^a^	*Pasteurella multocida*	[7]	AMPylates Rho GTPases (CDC42, TC10, RhoA and Rac1); cytotoxicity mediated by disruption of cytoskeletal regulation	crystal structure of the PfhB2 Fic domain is unavailable

^a^Crystal structures of dFIC, GS-ATase and the Fic domain of PfhB2 has not been solved.

^b^GS-ATase, DrrA and SelO exhibit non-Fic-mediated AMPylation.

#### 2.2.3. Pfhb2

PfhB2, a secreted virulence factor of *Pasteurella multiocida,* contains two Fic domains, Fic1 and Fic2, and shares a 64% amino acid sequence similarity with Ibp-A's Fic1 and Fic2 domains, respectively. Along with IbpA and VopS, PfhB2 forms a trifecta of bacterial Fic domain AMPylases that engage in host cell killing through AMPylation of the host Rho family GTPases. Similar to VopS and IbpA-Fic2*, in vitro*, PfhB2 AMPylates the conserved Tyr32 residue of Rho family GTPases (TC10, RhoB, RhoC and RhoG) and disrupts host cytoskeletal regulation, leading to gross anomalies in the cytoskeletal architecture.

Phylogenetic analysis revealed that these three AMPylases form a distinct ‘clade’ and branches away from other bacterial Fic domain proteins such as EcFicT, GS-ATase and HpFic. These observations indicate that these proteins may have evolved to target mammalian (host) proteins to facilitate their survival and promote virulence [7].

#### 2.2.4. *Bartonella* spp. effectors

The pathogenic *Bartonella* sp. mediates host cell subversion and pathogenicity by translocating Bartonella effector proteins (Beps) through its Type-IV secretion system (T4SS) into host cells. Some of these Bep effector proteins (table 1) contain an N-terminal Fic domain that catalyses AMPylation of host cell targets. BepA of *Bartonella henselae* was found to AMPylate yet-unknown targets having molecular weights of approximately 40 kDa and 50 kDa [60]. Bep1 of *Bartonella rochalimaea* AMPylates Rac1/2/3 and RhoG at Tyr32 causing cytoskeletal rearrangements that favour bacterial entry and survival, while Bep2 AMPylates mouse Vimentin, an integral component of intermediate filaments [43,45]. Target specificity of Bep1 (almost exclusively AMPylating Rac subfamily of Rho GTPases), as opposed to VopS (AMPylates Ras GTPase superfamily members indiscriminately), and IbpA-Fic2 (AMPylates almost all Rho but not Rac GTPase subfamily members). These salt bridges occur between (a) Asp117 and Lys119 of the extended flap region (a β-hairpin like structure that partially obstructs the active site and accommodates the Switch I region of its target through β-sheet augmentation), and (b) Lys116 and Asp124 of the T(K/Q)xD motif that forms a groove in Rac2 [44].

Recently, the Dehio group showed that the *Bartonella henselae* effector protein C (BepC) triggers actin stress fibre formation in HeLa cells through the activation of the RhoA GTPase signalling pathway by interacting with and relocating the nucleotide exchange factor GEF-H1 from its canonical location in the microtubules to the plasma membrane [59]. GEF-H1 activates RhoA by facilitating an exchange of GDP with GTP; this is followed by a series of downstream biochemical reactions that ultimately lead to the phosphorylation of myosin light chain (MLC), leading to actin stress fibre formation. The N-terminal BepC Fic domain interacts with GEF-H1 while the C-terminal BID (Bep intracellular delivery) domain anchors BepC to the plasma membrane [59]. Interestingly, the authors found that Fic domain-mediated AMPylation is not responsible for the interaction between BepC and GEF-H1 as a BepC quadruple mutant harbouring mutations in the active site (**H**146A, ***K***150A, **R**154A and **R**157A) of the Fic motif (**H**xFx***K***GNG**R**xx**R**) was able to trigger stress fibre formation as effectively as WT BepC. This observation indicates that this non-canonical BepC Fic domain (acidic Glu/Asp is replaced by a basic *Lys*) is involved in a non-catalytic role (protein–protein interaction) and is the first example to our knowledge where a bacterial FicD effector protein induces host cell killing without AMPylating any host target proteins [63,64].

**Figure 2.**
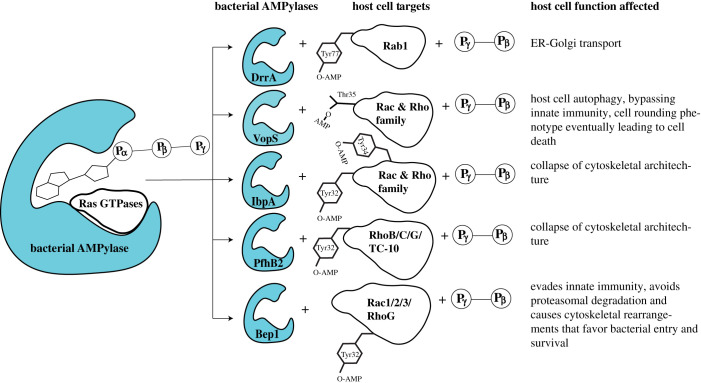
Targeting of Ras GTPase family members by bacterial AMPylases.

Together, these examples highlight that pathogenic bacteria employ protein AMPylation as a means to subvert host cell physiology, preferably targeting members of the Ras superfamily of GTPases (figure 2). Altering GTPase function through AMPylation enables these pathogens to evade host cell immune responses and/or affect downstream signalling pathways involved in cell maintenance. Future studies are expected to identify additional physiological targets of partially characterized bacterial FicD proteins that are translocated into host cells during infection where they may modify proteins to hijack host signalling (table 1).

## 3. Eukaryotic AMPylases

**Figure 3.**
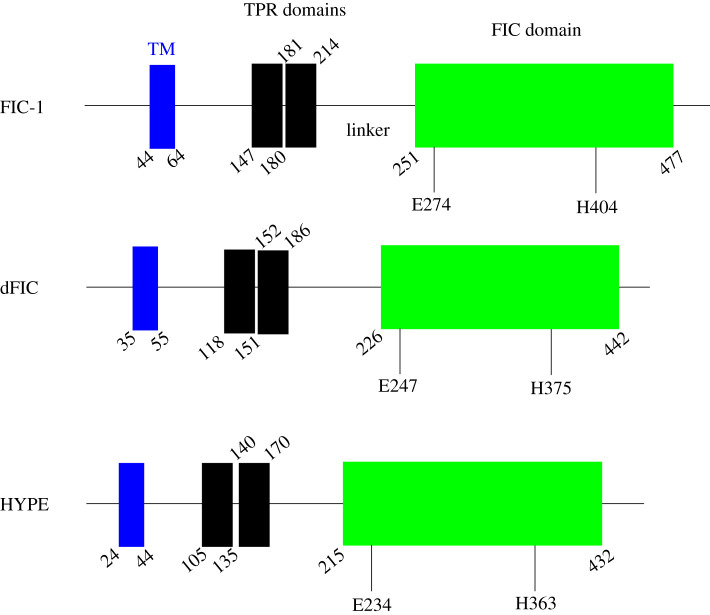
Domain organization schematic of metazoan AMPylases.

Most studies on metazoan AMPylases have focused on the human (FICD), *Drosophila melanogaster* (dFIC) and *Caenorhabditis elegans* (FIC-1) enzymes. These FicD-type AMPylases exhibit remarkable similarity in their structural architecture: an N-terminal transmembrane domain (TM) responsible for ER localization of these enzymes and anchoring to the ER luminal membrane, followed by one or two tetratricopeptide repeats (TPRs) involved in substrate recognition and specificity, and a C-terminal Fic domain linked to the TPRs by a less-conserved α-helical linker required for mediating allosteric conformational changes (figure 3) [5,6,56]. Extensive sequencing of eukaryotic genomes has revealed the presence of a single functional fic allele in most metazoans [65], probably acquired through multiple horizontal gene transfer events [29]. Recent work by the Tagliabracci laboratory has found that the human pseudokinase selenoprotein-O (SelO) efficiently transfers AMP to mitochondrial proteins [54]. This discovery of a non-ficD AMPylase shows that different folds can catalyse protein AMPylation. It is thus tempting to speculate that more metazoan AMPylases may be revealed in the future.

In the following segments, we review both FicD and non-FicD eukaryotic AMPylases and discuss similarities and differences in their structure and function.

### 3.1. Human SelO: a non-Fic AMPylase

#### 3.1.1. Structure

**Figure 4.**
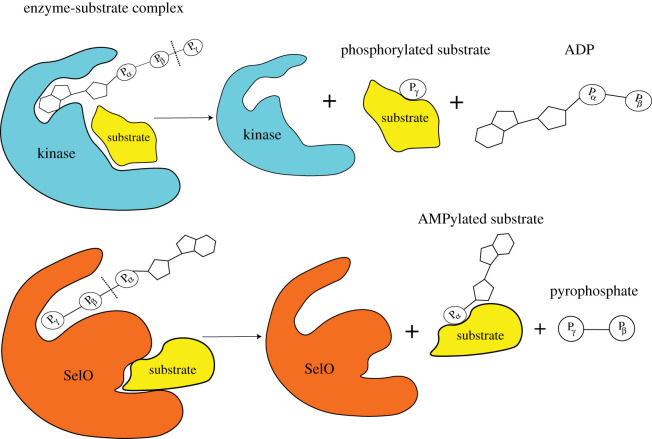
Flipped orientation of the ATP molecule in SelO. In the active site of a canonical kinase the adenine ring is buried deep in the catalytic cleft while the phosphates are exposed. Kinases transfer the distal phosphate (farthest from the adenine ring) onto its substrates (phosphorylated) with concomitant ADP release. In SelO, the orientation of the ATP molecule is flipped, with the phosphates buried deep in the binding cleft. SelO transfers the proximal phosphate (closest to the adenine ring) onto its target (AMPylated) with pyrophosphate being the other product of the AMPylation reaction. The dashed lines represent cleavage of the bond in a phosphorylation or AMPylation reaction.

Sreelatha *et al.* [54] recently discovered that the human pseudokinase, Selenoprotein-O (SelO) functions as an AMPylase. Pseudokinases are variants of canonical kinases that often contain key mutations in conserved kinase motifs, preventing ATP binding and hydrolysis [66]. Thus, these enzymes are proposed to mainly function as scaffolding agents [67,68]. A small subset of pseudokinases harbour compensatory mutations which reinstall or build a new active site that catalyses phosphorylation [69,70]. The three conserved kinase motifs in which pseudokinases often bear mutations are (a) the DFG motif, (b) the VAIK motif or (c) the HRD motif [66]. SelO carries a mutation of the catalytic Asp (in the HRD motif) that acts as a base in the catalytic loop and supports the phospho-transfer reaction [66]. Interestingly, the crystal structure of *Pseudomonas syringae* SelO bound to a non-hydrolysable ATP analogue AMP-PNP showed a flipped ATP molecule in the kinase-like fold. The kinase core of SelO adopted a typical kinase-like fold comprised of α-helices and β-sheets, but the γ-phosphate of AMP-PNP was found buried in between the two lobes of the kinase domain [54]. This is in contrast to nucleotide binding to canonical protein kinases where the α-phosphate remains buried, while the γ-phosphate is primed for phosphate transfer (figure 4). The binding site for the flipped nucleotide is formed by unique insertions in the β-sheet rich N lobe and the α-helical rich C lobe. Lys113 coordinates the γ-phosphate while Glu136 stabilizes this interaction. Arg176 and Arg183 also stabilize the γ-phosphate. Such interactions are also found in a canonical kinase but these interactions occur with the α-phosphate of ATP. Furthermore, Asp262 of the DFG motif acts a deprotonator of the phospho-acceptor hydroxyl on the protein side chain, and, together with Asn253, also coordinates the divalent cation Mg^2+^ which is required for stabilization of the α- and β-phosphates [54]. A comparative analysis of AMP-PNP binding conformations in SelO and a canonical kinase, Protein Kinase A (PKA), suggested that Asp262 of SelO acts similarly to Asp166 of PKA, except that the positioning of the SelO Asp262 facilitates a favourable conformation of the α-phosphate resulting in AMPylation, while Asp166 (PKA) facilitates a favourable conformation of the γ-phosphate leading to phosphorylation via ATP hydrolysis. Finally, the SelO structure also revealed that an intramolecular disulphide bond between Cys272 and Cys476 acts as an internal regulator of SelO AMPylase activity, a mechanism employed by multiple mitochondrial proteins [71].

*In vitro* autoradiography assays using recombinant human WT-SelO and radiolabelled ATP showed that SelO catalysed radiolabel transfer when using [*α*^−32^P] ATP, but not [*γ*^−32^P] ATP, as a reaction substrate, indicating that SelO is an AMPylase. Mutating the DFG motif in SelO resulted in a complete loss of AMPylation activity, suggesting that the observed AMPylation is SelO-specific. Similar to FicD-type AMPylases, SelO auto-AMPylates several tyrosine and serine residues and prefers ATP as a nucleotide substrate [54].

### 3.2. Functional implications of SelO AMPylation

Human SelO localizes to the mitochondria [72] and, like other SelO proteins, is involved in redox homeostasis [73]. To identify potential substrates AMPylated by SelO in the mitochondria, the Tagliabracci laboratory used biotin-17-ATP, *E. coli* extract and purified WT human SelO, and, following SelO-mediated *in vitro* modification of putative targets with biotin-17-AMP, retrieved biotinylated proteins using avidin. Comparative mass spectrometry revealed the presence of biotin-17-AMP-linked sucA, the bacterial homologue of α-keto dehydrogenase [74] and glutaredoxin (grx), a thioredoxin-like protein that catalyses deglutathionylation [75], as the top hits. Both of these proteins are involved in the regulation of redox homeostasis. Subsequent *in vitro* AMPylation assays confirmed SelO-mediated AMPylation of sucA at Thr405 and grx at Tyr13 [54]. Physiologically, SelO is required to protect *Saccharomyces cerevisiae* cells from H_2_O_2_-induced oxidative stress. SelO knockout (KO) yeast cells showed reduced cell viability under an oxidizing environment, which was rescued by the expression of WT SelO, but not the catalytically inactive SelO^D348A^. The AMPylated Tyr13 of grx lies within the conserved active site of this enzyme, which is proposed to influence the equilibrium between glutathionylated and deglutathionylated states of grx under oxidative stress conditions. Indeed, SelO KO yeast cells subjected to oxidized glutathione (GSSH), known to increase S-glutathionylation levels, showed a significant decrease in glutathionylation levels as compared to SelO KO yeast cells grown under normal growth conditions. This suggests that SelO inhibition of grx-mediated deglutathionylation is critical in maintaining this equilibrium, and a dysregulated equilibrium favouring enhanced deglutathionylation prevents the cell from implementing a cytoprotective response through the induction of S-glutathionylation [76].

### 3.3. Eukaryotic FicD AMPylases

Eukaryotic Fic AMPylases show little or no AMPylation activity under standard growth conditions. Overexpression of endogenous wild-type (WT) AMPylases has limited effect on physiological processes and recombinant WT AMPylases are poor *in vitro* AMPylators [6,36,40,52,56] but possess the catalytic ability to remove AMP from pre-AMPylated substrates (de-AMPylation) [77,78]. These AMPylases [5,6,56] are auto-inhibited by an inhibitory α-helix (α-inh) that sterically hinders ATP binding to the catalytic site, a mechanistic feature that is shared by a few of their bacterial counterparts [40]. The Glu residue in the conserved α-inh, represented by (S/T)xxx**E**(G/N) forms a salt bridge with an Arg in the catalytic motif HxFx(D/E)GN(G/K)Rxx**R**, locking enzymes in their ‘off’ inactive states. Mutating Glu to Gly relieves auto-inhibition and allows the transition to an ‘on’ state, where AMPylation activity is substantially enhanced [5,40]. On the other hand, mutating His to Ala in the catalytic Fic motif **H**xFx(D/E)GN(G/K)RxxR, results in complete ablation of AMP transfer, but not of ATP binding [30,36]. Both the constitutively active (Glu->Gly) and the catalytically impaired single mutants (His->Ala), as well as the double mutants (Glu->Gly/His->Ala) have served as workhorses for investigators studying the fundamentals of AMPylation.

HYPE and FIC-1 possess auto-AMPylation abilities in addition to target AMPylation. HYPE is modified at Thr183, Ser79 and Thr80, while FIC-1 is modified at Thr352 and Thr476 [6,52]. *Neisseria meningitides* AMPylase NmFic has also been shown to possess auto-AMPylation ability in *cis*. Auto-AMPylation (at Thr183 and Tyr188) of NmFic, leading to partial unfolding of the α-inh, is essential for its ability to modify target proteins [46].

#### 3.3.1. HYPE: *Homo sapiens*

Huntingtin Yeast Partner E (HYPE) was initially discovered in a yeast two-hybrid screen as one of several proteins (HYPA - HYPH) to interact with the N-terminus of Huntingtin; yet, its enzymatic functions remained unnoticed [79]. Years later, after the discovery that bacterial FicD proteins catalyse AMPylation reactions, HYPE was found to be the only human FicD protein and subsequently shown to possess both AMPylation and deAMPylation capabilities [5,51,53,77]. HYPE is predominantly found in the ER and the nuclear envelope continuum [52,80] but emerging evidence suggests a broader localization pattern of HYPE under certain circumstances.

#### 3.3.2. Structure of HYPE

**Figure 5.**
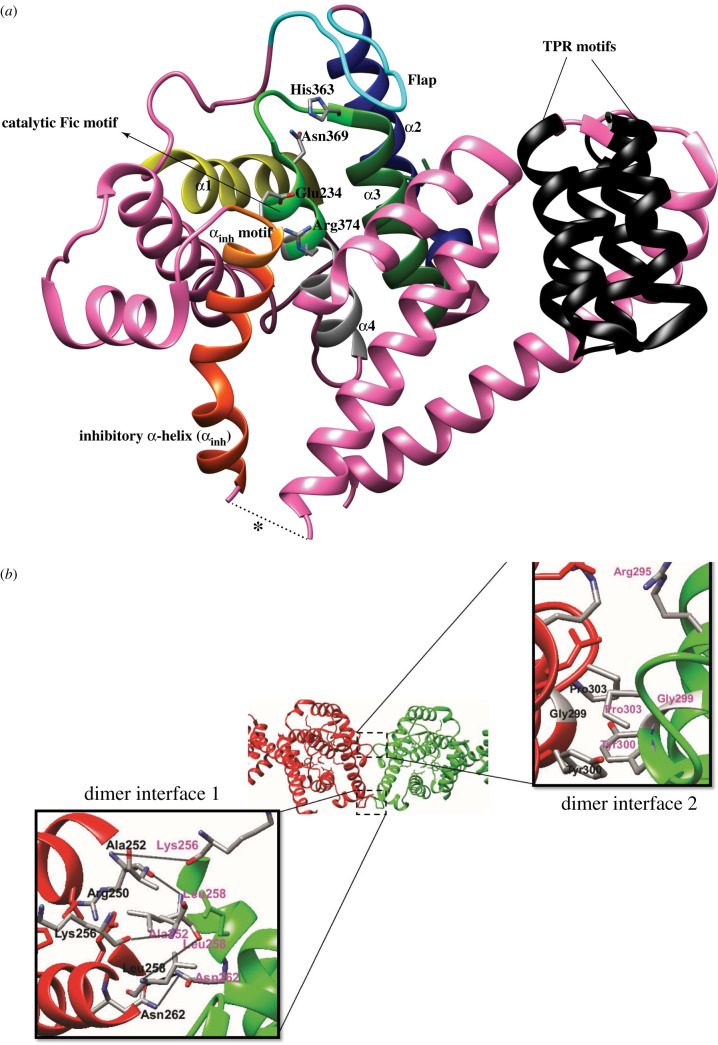
HYPE crystallizes as an asymmetric dimer. (*a*) A cartoon representation of a HYPE monomer. Key structural features are highlighted. Asterisk represents missing electron density for 6 residues within the linker. The same structural features are present in the other HYPE monomer but has not been shown here for clarity. (*b*) HYPE residues involved in the formation of interfaces 1 and 2 are labelled. The HYPE monomers are depicted in green and red. Black solid lines (Dimer Interface 1 inset) denote hydrogen bonds between residues. Residues making up Dimer Interface 2 interact weakly, mostly through Vander-Waal's and electrostatic interactions.

**Figure 6.**
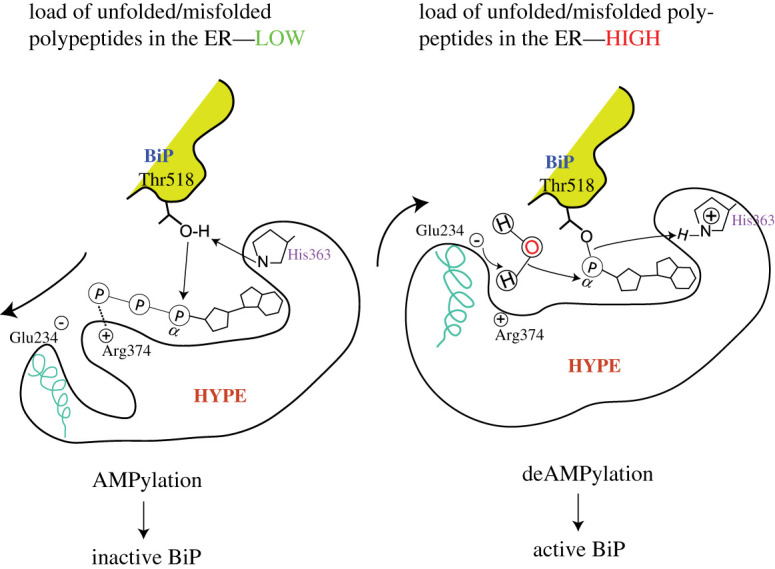
HYPE switching between AMPylation and deAMPylation states in response to ER conditions. HYPE AMPylates BiP when the unfolded protein load in the ER is low and deAMPylates BiP under conditions of stress that often increase unfolded protein load. AMPylated BiP cannot function as a chaperone and is pooled into a reservoir of non-functional BiP that can be activated under conditions of stress by deAMPylation. It is currently hypothesized that Glu234 mediates the switch between AMPylation and deAMPylation competent HYPE conformations. The bold arrows indicate the movement of α-inh that harbours Glu234. During AMPylation, Glu234 disengages from the catalytic site and allows the alignment of key residues in the catalytic FIC motif and Thr518 of BiP. When cells require functional BiP to tackle increasing loads of unfolded polypeptides, Glu234 engages with Arg374 and coordinates the attack of a water molecule (acting as a nucleophile) on the bond between Thr518 and AMP. The smaller arrows indicate a hypothetical electron transfer between various moieties involved in the proposed catalytic mechanism. This figure is adapted from Preissler *et al*. [77].

HYPE consists of a single transmembrane domain (residues 24–44), two TPR domains TPR1 (residues 105–135) and TPR2 (residues 140–170), and the conserved, catalytic Fic domain (residues 216–432) joined to the TPR motifs by a short linker (residues 170–215) (figure 3). HYPE is predominantly N-glycosylated at Asn275, which is responsible for its ER localization [52]. The TPR motifs, which are classic protein-protein interaction domains [81], are implicated in contributing to HYPE's target recruitment [82]. The crystal structure of HYPE*_Δ_*_102_ revealed that HYPE is mainly composed of α-helices (figure 5*a*). The Fic domain of HYPE is structurally similar to the Fic domains of VopS, IbpA, FIC-1 and dFIC. The Fic core comprises two highly conserved structural features: (a) the catalytic loop and (b) the flap (figure 5*a*) [83]. The catalytic core contains the highly conserved Fic motif HPF(I/V)DGNGRT(S/A)R, while the flap (residues 311–324) is involved in positioning of the target residues. HYPE, unlike bacterial AMPylases VopS and IbpA, possesses an auto-inhibitory helix (α-inh) containing the inhibitory motif (T/S)V(A/G)IEN (figure 5*a*) [5,40]. HYPE crystallizes as an asymmetric dimer with the dimer interface formed exclusively through interactions between Fic domain residues (figure 5*b*). Leu258 is critical for HYPE dimerization as the HYPE^L258D^ single mutant purified as monomer [5]. The Apo-HYPE crystal structure showed Glu234, critical for auto-inhibition, positioned in close proximity to the catalytic loop. Crystal structures of ADP bound to WT HYPE and ATP bound to the HYPE^E234G^ variant revealed accommodation of the α-phosphate by the GNG anion hole (residues 368–370) through hydrogen bonds. A Mg^2+^ ion in the ATP-HYPE^E234G^ structure coordinates with the conserved Asp367 to bridge the α- and β-phosphates. The conserved Arg374 at the C- terminus of the Fic motif forms hydrogen bonds with the ribose ring and mediates binding of the γ- phosphate. The α-inh in apo and ADP-bound variants of HYPE obstructs the engagement of β- and γ- phosphates as it competes with the Arg374/γ-phosphate interaction, resulting in a non-productive orientation of the α-phosphate [5,40]. This partial obstruction of the ATP binding site has also been observed in the FicD proteins of *Neisseria meningiditis* and *Shewanella oneidensis*, where N- and C-terminal extensions of the α-inh protrude into the catalytic Fic domain [40]. This inhibition was released when Glu234 was mutated to Ala [5]. AMP transfer in the HYPE^E234G^ variant is catalysed by the conserved His in the Fic motif, which acts as a base to attack the nucleotide's phosphodiester bond, resulting in AMP transfer and the concomitant release of a pyrophosphate group (PP_i_) (figure 1*b*). While some kinetic studies point to a sequential reaction where auto-AMPylation is followed by target AMPylation, other studies hypothesize a substrate-assisted attack (figure 6), where the conserved His functions as a proton acceptor (accepts a proton from a substrate Thr/Tyr), resulting in a subsequent nucleophilic attack by these residues on the α-phosphate of ATP [35].

WT HYPE acts primarily as a deAMPylase [77,84,85] and the deAMPylation mechanism proposed by David Ron's group [77] suggests that Glu234 is actively engaged (the positively charged Arg374 holds the negatively charged Glu234 side chain in proximity to the active site) in coordinating a water molecule which acts as a nucleophile and attacks the phosphodiester bond between the hydroxyl group of a target residue (BiP Thr518) and the AMP moiety attached to the active site of HYPE (figure 6). Consequently, the AMP moiety is removed from the catalytic site and His363, which in the AMPylation reaction deprotonates and hence, primes the hydroxyl group of Thr518 to act as a nucleophile, now protonates the leaving Thr518 group [77]. Thus His363 is an active site residue that mediates both AMPylation and deAMPylation reactions, albeit in a polar opposite fashion.

#### 3.3.3. HYPE function

HYPE and other metazoan AMPylases are tightly regulated. Knockdown of HYPE in HeLa cells subjected to ER stress-inducing agents resulted in reduced viability as cells failed to cope with increased ER stress [52]. Similarly, *FIC^−/−^* Chinese hamster ovary cells (CHO) show a delay in UPR activation under ER stress-inducing conditions compared to WT CHO cells. HYPE plays a positive role in mediating an unfolded protein response (UPR) in the ER via the ATF-6 and PERK branches of the UPR pathway. This transcriptional upregulation of UPR genes is mediated by *cis*-acting UPR elements found in the HYPE promoter sequence [52]. On the other hand, overexpression of a constitutively active HYPE^E234G^ triggers caspase-dependent apoptosis leading to elevated levels of cytotoxicity and eventual cell death [80].

**Figure 7.**
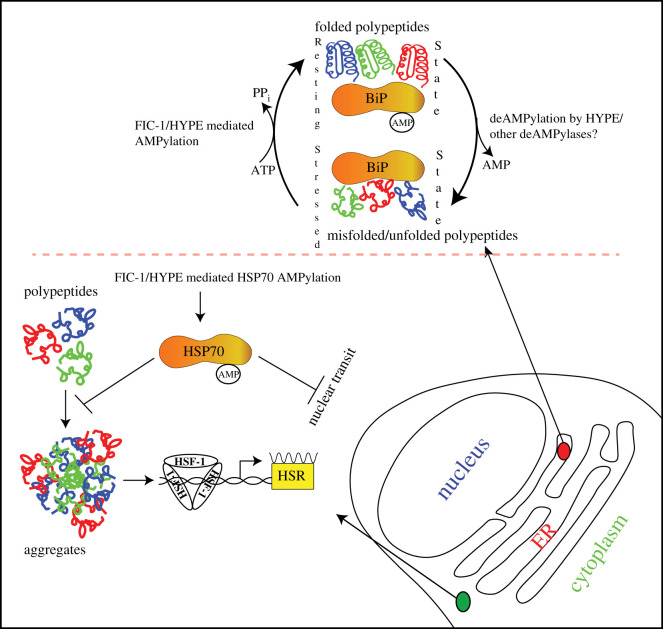
Consequences of HYPE functions in the cytoplasm and ER. The arrows pointed outwards from the green and red zoom-in circles depict events regulated by HYPE mediated AMPylation in cytoplasm and ER, respectively.

In the ER, HYPE regulates the function of the ER-resident HSP70 family chaperone BiP through cycles of AMPylation and deAMPylation (figure 7). Molecular docking studies revealed that the WT HYPE dimer binds BiP^T229A^ (capable of binding but not hydrolyzing ATP) in a 2 : 2 stoichiometric ratio. The nucleotide binding domain of BiP binds to the TPR domains of HYPE through extensive hydrogen binding [82]. WT HYPE behaved as a weak AMPylator while HYPE^E234G^ was efficient in catalysing both auto- and target AMPylation. BiP is modified at two sites: Ser365/Thr366, and Thr518 [52]. AMPylation of BiP at Ser365/Thr366, located in the nucleotide binding domain (NBD), was first described by the Mattoo and Orth groups. The NBD of BiP is involved in both ATP binding and hydrolysis functions of BiP [86]. Thus, AMPylation of BiP residues (Ser365/Thr366) located in the NBD could affect ATPase function of BiP. Indeed, HYPE^E234G^ and, to a lesser extent, WT HYPE-mediated AMPylation of BiP increased its ATP hydrolysis rate compared to unmodified BiP, most likely due to allosteric modulation of the ATP binding active site [52].

In agreement with Sanyal *et al*.'s *in vitro* study [52], Ham *et al*. found Thr366 of BiP to be AMPylated *in vitro* by the *Drosophila melanogaster* Ficd enzyme, dFic [56]. However, while Sanyal *et al*. proposed that BiP AMPylation positively regulates chaperone function by significantly enhancing ATPase function of BiP that allows the cell to mount a robust BiP mediated activation of UPR pathways [52], Ham *et al*. observed that BiP AMPylation correlates with reduced BiP ATPase activity and AMPylated BiP is an inactive chaperone that is not capable of upregulating/activating downstream UPR pathways [56]. Importantly, Sanyal *et al*. conducted assays with full-length WT human BiP while Ham *et al*. used a truncated construct (residues 27–417) [56] that consisted of the BiP ATPase domain (residues 27–407) and an additional linker (residues 408–417) [87]. Sanyal *et al.* argue that such truncated constructs could be responsible for altered ATPase activity of AMPylated BiP constructs, although how such truncations affect the correlation between AMPylation and ATPase activity has not yet been elucidated.

Preissler *et al.* [88] showed that HYPE also AMPylates BiP at Thr518 *in vitro* and in CHO cells. Thr518 is located in the substrate binding domain (SBD) of BiP and is stabilized by intramolecular polar interactions in apo or ADP bound BiP [89]. ATP-bound BiP, however, lacks these interactions and the residue is therefore free to interact with the active site of HYPE. BiP AMPylation on Thr518 reduces the ATPase-dependent protein folding turnover rate but does not prevent client, co-chaperone or nucleotide binding [52,84,88,90]. BiP AMPylation also impairs the formation of inactive structured BiP oligomers [84]. The current consensus model suggests that unstressed cells maintain a pool of inactive AMPylated or oligomeric BiP. When the cells sense an increase in the load of unfolded polypeptides, this inactive pool of BiP is activated by rapid de-AMPylation or monomerization. This active BiP population then readily engages in chaperoning activities enabling the cell to mount a stress response in the ER. Once proteostasis is restored, HYPE will AMPylate and thereby inactivate a significant fraction of BiP again. Simultaneously, a fraction of non-AMPylated BiP will oligomerize and be excluded from active chaperoning duty [91–93]. Experiments using ATPase-deficient BiP^T229A^ and BiP^E201G^ mutants, or nucleotide-free BiP, demonstrated that HYPE preferentially binds and locks the ATP-bound ‘committed-to-hydrolysis' BiP state as opposed to a relaxed ADP-bound conformation of BiP (attained after ATP hydrolysis and concomitant release of folded protein) [88]. The crystal structure of AMPylated BiP adopts a domain-docked conformation, and the NBD of AMPylated BiP attains a similar conformation to ATP bound WT BiP. The distal loops of the SBD*β* subdomain, harbouring the AMPylation site Thr518, also exhibits substantial conformational changes to accommodate the AMP moiety [89].

Sanyal *et al.* recently approached the physiological impact of BiP Ser365/Thr366 versus Thr518 AMPylation by performing advanced *in-silico* molecular docking experiments to generate various conformations of HYPE-BiP that could dissect the role of NBD and SBD in HYPE-mediated BiP modifications. The orientation of the enzyme active site seemed to be critical for accommodating either the NBD or the SBD of BiP [82]. In the first orientation, the active site interface formed between the NBD and HYPE favoured AMPylation at Ser365/Thr366 and precluded the need for SBD to interact with NBD. In accordance with such an observation, HYPE^E234G^AMPylated the isolated BiP_NBD_ but not the isolated BiP_SBD_. The second orientation favoured AMPylation of Thr518, and HYPE formed active-site interfaces with both domains of BiP, indicating that AMPylation of Thr518 in the SBD is influenced by allosteric cross-talk between both domains [82]. Thus, AMPylation at Ser365/Thr366 acts antagonistically to AMPylation at Thr518, and such modifications in tandem could act as internal regulators of AMPylation-dependent BiP function.

Preissler *et al.* carried out several experiments to further investigate whether WT HYPE could act as a deAMPylase, and to determine how the transition between the AMPylated and deAMPylated states of BiP occurs [77]. They reported that WT HYPE deAMPylates hamster BiP *in vitro* and overexpression of WT HYPE was not able to restore BiP AMPylation in *FICD^−/−^* CHO cells [77]. Overexpression of WT HYPE prevented the accumulation of inactive, AMPylated BiP, thereby de-repressing its function [77]. HYPE was found to act as a deAMPylase and the deAMPylation function was specific to full length WT BiP, as neither the isolated SBD nor the AMPylation-deficient BiP^T518A^ was deAMPylated. HYPE^E234G^ was not able to catalyse de-AMPylation and did not prevent WT HYPE-mediated BiP de-AMPylation *in vitro*. Transient overexpression of HYPE^E234G^ in CHO cells resulted in increased UPR signalling and AMPylation of BiP, which was attenuated when WT HYPE was co-expressed [77]. This work established HYPE as a bi-functional enzyme that catalyses both BiP AMPylation and deAMPylation.

The switch between AMPylated and deAMPylated BiP conformations depends on the oligomeric state of HYPE and the metal ion required to coordinate ATP or AMP positioning in HYPE's active site [85]. Such internal structural regulation of an AMPylase function had been previously observed in *Clostridium difficile*, where monomeric CdFic exhibited increased auto-AMPylation [48]. Strictly monomeric AMPylation competent HYPE^L258D/E234G^ AMPylates BiP *in vitro* and *in vivo* when overexpressed in *FICD^−/−^* cells, as opposed to dimeric WT HYPE [85]. DeAMPylation activity of the monomeric HYPE^L258D^ and partially monomeric HYPE^G299S^ mutants is reduced approximately two-fold as compared to WT HYPE. In addition, AMPylated BiP binds more tightly to dimeric HYPE as compared to monomeric HYPE. Together, this data suggests that monomeric HYPE mutants possess enhanced AMPylation function, and a slightly but significantly repressed deAMPylase function [85]. Recent work by Veyron *et al.* has shown that the WT HYPE-mediated BiP deAMPylation can be fine-tuned by modulating the Ca^2+^/Mg^2+^ ratio *in vitro* [47]. Increasing this ratio or removing Mg^2+^ completely from the reaction mixture decreases the deAMPylation efficiency of WT HYPE. Conversely, WT HYPE efficiently catalysed BiP deAMPylation in the presence of only Mg^2+^ [47]. This observation, coupled with similar results observed for the bifunctional *Enterococcus faecalis* Fic (EfFic), led the authors to propose a divalent metal ion differential as an alternative (or additional) mode of regulation for the displacement of Glu234 (inherent conformational flexibility) [47]. These results raise the possibility that the FIC domain may act as an enzymatic calcium sensor, and prompt us to ask whether HYPE can respond to diffusible signals, such as a drop in ER calcium levels under stress conditions.

The crystal structure of apo-HYPE suggests that hydrogen bonds involving Lys256 and Glu242 link the dimer interface with the enzyme's active site and impinges on the auto-inhibitory Glu234, as both K256S and E242A mutants formed dimers and AMPylated BiP [5]. Furthermore, combining K256S with an L258D mutation rendered HYPE monomeric and led to a further increase in BiP AMPylation. These observations indicate that allosteric cross talk between the dimerization interface and the catalytic active site, mediated by linker residues, leads to de-repression of AMPylation function [77,85]. The α-phosphates of the nucleotides (ATP/AMPPNP) bound to the AMPylation-efficient HYPE variants (HYPE^L258D^ and HYPE^L256S^) were observed to be in an AMPylation competent state, as opposed to WT HYPE where the α-phosphate was in an AMPylation non-competent state and was unable to coordinate the divalent cation Mg^2+^. These mutants bound ATP/AMPPNP in a similar fashion compared to HYPE^E234G^ [5,85]. The inhibitory Glu234 in these mutants was displaced from its position in the apo-state and was thus rendered incapable of forming a salt bridge with Arg374. Consequently, the catalytic site is relieved from steric hindrance as is observed in the apo- or ATP bound WT HYPE structures [85]. ATP acts as an external allosteric modulator of HYPE function by increasing the HYPE monomer to dimer ratio in a concentration-dependent manner, while ADP acts as an antagonist, pushing the equilibrium towards dimer formation [85]. Thus, the functions of both BiP and HYPE are tightly regulated by intrinsic structural changes (oligomerization) as well as external modulators that dictate the propensity of BiP to be modified by HYPE.

## 4. HYPE: target selection

Before the discovery of HYPE-mediated BiP AMPylation, initial work using GST-tagged full- length HYPE, as well as the His_6_-tagged isolated Fic domain of HYPE (181–458), indicated that, similar to VopS and IbpA, HYPE may AMPylate CDC42, Rac1 and RhoA *in vitro* [7,36,40]. However, further experiments using His_6_-tagged HYPE_45-458_ (E234G) or HYPE_103-445_ (E234G) failed to confirm Rho GTPase AMPylation, suggesting that the Rho family of GTPases are not physiological targets of HYPE [52]. Several studies have indicated that HYPE variants lacking the TPR domains are more promiscuous; indeed, HYPE_187-437_ AMPylates recombinant HSP40, HSP70 and HSP90 *in vitro* [51].

**Figure 8.**
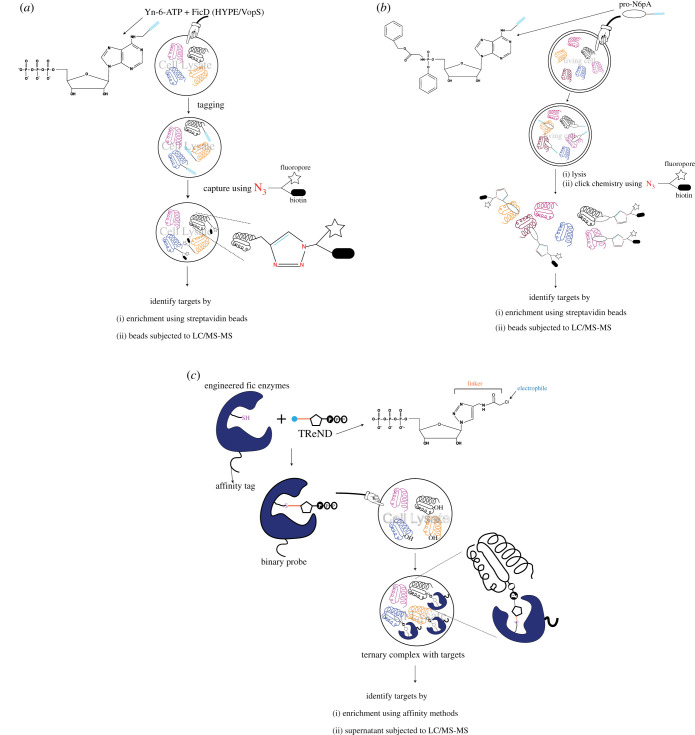
Pictorial representation of approaches used to capture and identify FicD targets. Panels (*a*) and (*b*) represent approaches that modify targets using synthetic nucleotides followed by a click-chemistry-based capture of modified targets while in (*c*) the enzyme is modified using TReND and forms a binary complex that then AMPylates target proteins.

Aside from BiP, a significant set of putative HYPE targets were identified in peptide array and chemical proteomics screens by several groups. Broncel *et al.* identified 25 substrates of HYPE in HEK293 cells using a two-step chemo-enzymatic tagging strategy (figure 8*a*) that installed an AMP-biotin instead of an AMP group on AMPylated proteins [49]. Apart from BiP, several other proteins involved in regulating transcription (eEF1a1 [94], E1F2AK2 [95]), cytoskeletal development (TUBB [96]), ATP synthesis (ATP5A1 [97], ATP5B [98]) and the ubiquitin-proteasome complex (UBAP2 L [99]) were found [49]. Histones H1–H4 were also found to be modified by HYPE *in vitro* [80]. A major caveat of this strategy is that the AMPylation reaction relied on supplementing cell lysates with HYPE^E234G^ and Yn-6-ATP. Thus, the physiological sub-compartmental context was lost with HYPE^E234G^ potentially modifying proteins that, in an intact cell, would be secluded from its reach. Kielkowski *et al.* circumvented this problem by chemically synthesizing an adenosine pronucleotide probe (pro-N6pA) that demonstrated higher cell permeability than commercially available ATP analogues and also bypassed kinase- mediated phosphorylation of the nucleoside moiety [100]. This precursor is metabolically converted to N6pATP and is used in cellular AMPylation reactions. In their pioneering study, the Sieber laboratory treated HeLa cells with pro-N6pA, labelled the N6p-AMP-containing proteins in a click-chemistry reaction with biotin-azide, and affinity enriched the modified proteins by avidin pulldown (figure 8*b*). Mass spectrometry-based protein identification revealed that Cathepsin B (CTSB) is a novel target of HYPE, with HYPE^E234G^ AMPylating CTSB on the cysteine residues that lie within its conserved catalytic site [101]. CTSB is a lysosomal cysteine protease involved in protein catabolism and antigen processing [102], suggesting that HYPE may play a regulatory role in these processes. Further proteomic profiling using this method in three different cancer cell lines identified a total of 58 AMPylated proteins, most of which were proposed to not be modified by HYPE. This leads to the hypothesis that there might be additional yet-to-be-identified AMPylases involved in the regulation of different metabolic and proteostasis pathways [100]. Chemical-proteomic profiling of a neuroblastoma cell line, SH-SY5Y, under ER stress conditions led to the identification of 145 AMPylated proteins, indicating that AMPylation may play a unique role in the central nervous system. Further analysis of AMPylation profiles in neuronal progenitor cells (NPCs) revealed that the majority of AMPylated proteins were involved in cytoskeletal remodelling and vesicular transport, indicating that AMPylation may be involved in neuronal development [100]. The authors further modified the protocol to obtain live cell imaging of protein AMPylation using a slightly modified probe (pro-N6pAazA) that binds to potential endogenous targets; subsequently, the targets are either linked to PEG-biotin (for LC-MS/MS based target identification) or coupled to a fluorophore that can be stained for and visualized using fluorescence microscopy [50]. This protocol preserves the integrity of the cell and provides relevant physiological information about the sub-compartmental context of AMPylation. The authors, in addition to targets identified using the pro-N6pA approach, found CTSC and CTSZ (cysteine cathepsins), ABHD6 (a lipase), ACP2 (a lysosomal acid phosphatase), PNPLA3 (catalyses coenzyme-A mediated acylation) and TPP1 (a serine protease) to be modified [50]. Live cells tolerated the probe well and time-lapse fluorescent imaging in fibroblast cells revealed directional transport of AMPylated proteins across processes extending out from the ends of the cell bodies and implicates AMPylation in polarization of fibroblasts [50]. Such a tool can thus be used to study real time AMPylation inside cells subjected to various conditions of stress.

Recently, Gulen *et al*. [103] developed a novel strategy for capturing AMPylated targets. This strategy is based on the covalent attachment and activation of a nucleotide co-substrate inside the active site of the enzyme. The resulting binary probe (enzyme-nucleotide complex) subsequently captures modified targets (figure 8*c*) [103]. This strategy required the strategic placement of a Cys residue close to the ATP binding pocket of FicD enzymes (Ile3755/Asn111/Glu404 was mutated to Cys for IbpA/BepA/HYPE, respectively). Next, synthetic thiol-reactive nucleotide derivatives (TReNDs) were designed to stably attach to the Cys residue of the AMPylase for optimal reactivity. These binary probes (Fic^TREND^) were then incubated with HEK293T cell lysates where they formed a stable ternary complex with endogenously modified targets. Unlike other methods, this technique avoids competition with endogenous ATP, as the enzyme's ATP binding site is irreversibly occupied by a TReND and thus can no longer accommodate unmodified ATP [103]. Using this approach, the Itzen group identified several new targets of IbpA, BepA and HYPE in addition to previously known targets like CDC42 (IbpA) and BiP (HYPE). For HYPE, the most enriched hits were eEF1A2 (transcription factor), UMPS (involved in UMP biosynthesis), CDK4 and CDKN2A (proteins involved in cell cycle progression), SUGT1 (a protein probably involved in ubiquitination and proteasomal degradation), MAPK1IP1 L (regulates cell differentiation and proliferation) and EIF3D (translation initiation factor) [103].

Sanyal *et al*. recently showed that HYPE also AMPylates α-synuclein *in vitro* [53]*.* HYPE binds and AMPylates α-synuclein at residues Thr33, Thr54 (located in the amphipathic N-terminus of the protein) and Thr75 (located in the central hydrophobic NAC domain). WT α-synuclein disrupts the lipid bilayer integrity through its interaction with membrane surfaces, and sequesters aggregated α-synuclein aggregates in extracellular space, leading to neurotoxicity. AMPylated α-synuclein displayed reduced membrane permeabilization and disruption of membrane bound vesicles, suggesting that HYPE-mediated AMPylation of α-synuclein may attenuate neurotoxicity [53]. *In vitro* assays using Thioflavin T to monitor aggregation kinetics of mouse WT and AMPylated α-synuclein showed that the AMPylated protein displayed a compromised ability to form fibrils as compared to WT α-synuclein aggregation, which reached saturating levels under assay conditions. Furthermore, transmission electron microscopy (TEM) analysis of amyloid-like fibrils formed by AMPylated α-synuclein as opposed to WT α-synuclein revealed distinct morphological differences [53]. Thus, AMPylation modulates α-synuclein pathology through altering fibril structure-associated toxicity, and may be triggered as a neuroprotective response to elevated levels of α-synuclein aggregates. Although *in vivo* relevance of these findings are yet to be conclusively ascertained, HYPE localization in the dopaminergic neurons of the *substantia nigra* of WT rats [53] raises the possibility that HYPE may indeed AMPylate α-synuclein in dopaminergic neurons, which are predominately affected by α-synuclein aggregation in Parkinson's disease (PD).

### 4.1. dFIC (CG9523): *Drosophila melanogaster*

dFIC is the *Drosophila melanogaster* HYPE orthologue. Similar to HYPE, dFIC is glycosylated at Asn288, releasing the functional protein into the ER lumen. dFIC also localizes on the surface of glia cells, and is enriched in the capitate endings that these cells use to interact with synaptic endings of photoreceptor synapses [55]. To identify potential targets of dFIC, the Krämer laboratory performed pull-down assays that used lectin concanavalin A to enrich glycoproteins in cell lysates followed by *in vitro* AMPylation using radiolabelled ATP and the constitutively active dFIC E247G mutant. MS analysis revealed BiP as the major dFIC target, which was confirmed by performing *in vitro* AMPylation assays with purified recombinant dFIC and BiP. As observed for HYPE, the catalytically inactive dFIC^H375A^ lacked AMPylating ability, whereas WT dFIC showed weak levels of AMPylation [56]. As observed in human cells, fly cells showed a rapid decline in AMPylated BiP at 30 min post-ER stress induction [56], suggesting that BiP deAMPylation upon ER stress provides the cells with heightened levels of active BiP sufficient to reduce the burden of unfolded proteins.

dFIC AMPylates BiP at Thr366, a residue close to the ATP binding site; the authors reasoned that attaching an AMP moiety to this residue may influence BiPs' ATPase activity. However, mutant BiP^T366A^ still hydrolysed ATP efficiently [56]. This observation suggests that, similar to HYPE-mediated BiP AMPylation, the modification of BiP by dFIC does not affect BiP's ATPase function. dFIC also shows a preference for AMPylating an ATP-bound but substrate-binding-deficient BiP conformation [56,104].

A hyperactive dFIC double mutant that lacks the ability to dimerize (dFIC^E247G/I271D^), showed significantly reduced levels of auto-AMPylation as compared to dFIC^E247G^. However, substrate AMPylation (BiP^T229A^) using this double mutant and each of the single mutants (dFIC^E247G^ and dFIC^I271D^) showed that both single mutants were able to AMPylate the substrate, with the double mutant exhibiting target AMPylation levels several-fold higher than either of the single mutants alone [78]. These experiments demonstrate that loss of oligomerization leads to enhanced BiP AMPylation. Increased substrate AMPylation of BiP by a dFIC monomer may result from a tighter interaction of the monomer with BiP or an increase in the transfer rate of AMP to the BiP side chain. Alternatively, intra-molecule interactions between the two monomeric dFIC arms in WT dFIC could negatively regulate auto-AMPylation or ATP binding kinetics. It is tempting to speculate that relieving auto-inhibition and inhibiting dimerization acts synergistically to enhance the function of dFIC, although more biophysical studies evaluating any possible allosteric crosstalk between the catalytic Fic motif and the dimerization interface need to be performed.

Recombinant dFIC efficiently de-AMPylates AMPylated BiP^T229A^ *in vitro* and in cell lysates [77,78]. Unlike AMPylation, deAMPylation activity of dFIC is not affected by monomerization, as the strictly monomeric mutant enzyme dFIC^I271D^ is able to deAMPylate AMP-BiP^T229A^ *in vivo* and *in vitro*. Over expression of dFIC^E247G^ in a *fic*-null background is lethal but well tolerated in a wild-type background. Furthermore, eye-specific expression of dFIC^E247G^ resulted in a rough eye phenotype characterized by severe morphological defects in eye substructures in *fic*-null but not in WT flies [78]. These results indicate that harmful dFIC^E247G^ mediated hyper-AMPylation is counterbalanced by WT dFIC's deAMPylation activity.

Flies harbouring a loss-of-function mutant Fic^55^ allele, albeit viable and fertile, are blind and fail to activate postsynaptic neurons [55]. Vision can be restored by introducing WT dFIC, but not the catalytically inactive dFIC^H375A^ transgene [55]. dFIC function is also involved in histamine metabolism and neurotransmitter recycling processes [55], however, the mechanistic link between AMPylation and neurotransmitter recycling remains to be established. *fic*-null flies and flies harbouring AMPylation-deficient BiP^T366A^ further show defects in rhabdomeres, eye substructures linked to photoperception, when subjected to constant light (LL), implying that AMPylation regulates the structural and morphological integrity of the fly eye [57]. Furthermore, BiP AMPylation is essential for retaining visual acuity under LL conditions: Unlike WT flies, *fic*-null flies and flies harbouring the BiP^T366A^ mutant lack the reserve, inactive pool of AMPylated BiP. Upon LL conditions that induce ER stress, WT flies can quickly respond by deAMPylating the reserve pool of AMPylated BiP, thereby allowing the chaperone to engage in the active refolding of misfolded polypeptides. However, flies that are deficient in BiP AMPylation experience a delayed UPR activation [57]. This is because the cell needs to transcriptionally upregulate BiP production to cope with the rising levels of misfolded aggregates as (a) there is no reserve pool of AMPylated BiP and (b) a large fraction of active BiP is already engaged in housekeeping functions.

### 4.2. FIC-1: *Caenorhabditis elegans*

The HYPE orthologue FIC-1 is the sole member of the Fic domain family in the nematode *C. elegans*. Crystal structures of FIC-1 and FIC-1^E274G^ (corresponding to HYPE^E234G^) showed significant similarities in the core α-helical structure of the Fic domain, including the α-inh helix, which contains the auto-inhibitory glutamate at position 274. The TPR domains are similarly stacked (with respect to HYPE) while the linker region is structurally less similar (figure 3) [6]. FIC-1 crystallizes as an asymmetric dimer, and Val292 and Ile298 were found to mediate interaction between the two monomers at their respective dimerization interfaces. FIC-1 dimerization and its AMPylation activity are interlinked as the FIC-1^E274G/I298D^ double mutant showed significantly reduced substrate AMPylation but only slightly reduced auto-AMPylation [6]. This is in contrast to the behaviour of the dFIC^E247G/I271D^ double mutant, which exhibited reduced auto-AMPylation but very high levels of substrate AMPylation [78]. More biophysical studies are required to evaluate how structural differences between these orthologues result in such functional differences. FIC-1 is found in low levels throughout the worm body and during all its developmental stages. Fluorescence microscopy of embryos expressing FIC-1 under a strong heat shock promoter, following heat shock and subsequent FIC-1 overexpression, revealed a notable accumulation of the enzyme at the nuclear/ER interface, similar to intracellular HYPE localization. A small FIC-1 population was also found in the cytoplasm [6].

**Figure 9.**
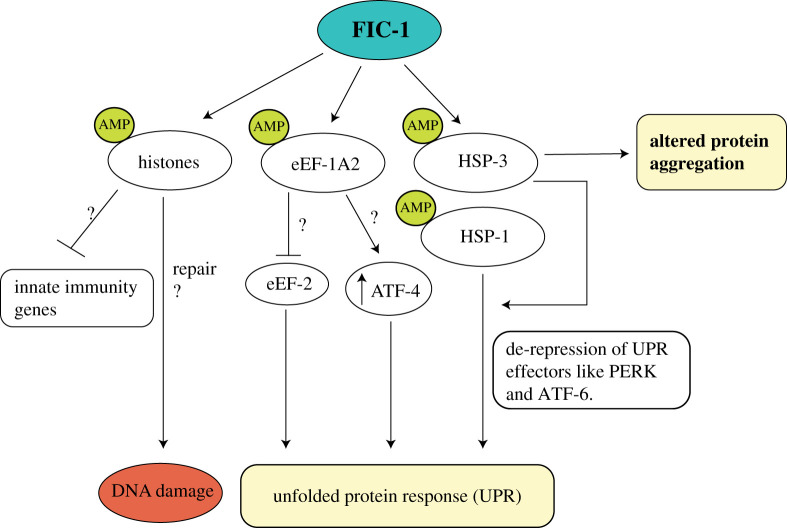
FIC-1 targets and their role in various physiological processes.

FIC-1 targets were identified using a click-chemistry based approach where worm lysates were spiked with recombinant FIC-1 and N^6^-propargyl-ATP as a nucleotide substrate. This was followed by the covalent coupling of a biotin-azide handle to putative AMPylated target proteins and their subsequent pull down using streptavidin beads (figure 8*a*) [6]. LC-MS/MS analysis of these samples identified the HSP70 family chaperones HSP-1 and HSP-3, the translation elongation factors eEF-1A, eEF-2, and eEF-1G, and the histones H2 and H3 as FIC-1 targets (figure 9). HSP-1 (orthologue of human cytosolic HSP70) and HSP-3 (orthologue of human BiP), were found to be AMPylated on residues Thr347 and Thr176, respectively [6]. Mutating the residues orthogonal to human BiP Ser365/Thr366 and Thr518 in *C. elegans* HSP-1 and HSP-3 (Ser370Ala/Thr371Ala and Thr523Ala) did not prevent HSP-1 and HSP-3 *in vitro* AMPylation by FIC-1^E274G^ or HYPE^E234G^ [6]. Truncated variants of FIC-1^E274G^ (FIC-1^258-508^ and FIC-1^134-508^) exhibit high levels of auto-AMPylation and auto-GMPylation, and moderate levels of auto-CMPylation and auto-UTPylation, indicating a high degree of promiscuity when choosing nucleotide triphosphates [6]. Like WT HYPE, WT FIC-1 showed very low levels of auto and target AMPylation while the catalytically inactive FIC-1^H404A^ (analogous to HYPE^H363A^) was unable to AMPylate target proteins. Interestingly, neither *fic-1* deficiency nor the expression of constitutively active FIC-1^E274G^ from the endogenous *fic-1* promoter affected worm development in the presence of tunicamycin-induced ER stress [6].

The inducible expression of *C. elegans* FIC-1^E274G^ in *Saccharomyces cerevisiae* leads to growth arrest and cell death [51]. FIC-1^E274G^ expression in this artificial set-up resulted in AMPylation of the cytosolic HSP70 family chaperone Ssa2 (figure 7), the induction of an unregulated heat shock response and the formation of protein aggregates in the ER and the cytoplasm. Importantly, over-expression of HYPE^E234G^ in human cells also triggers a heat shock response and the formation of nuclear HSF-1 punctae, thus showing the value of purely artificial set-ups to increase our understanding of physiologically relevant processes.

Truttmann *et al.* investigated the role of AMPylation in protein aggregation dynamics and associated toxicity; a hallmark of neurodegenerative diseases (NDs) [58]. Several NDs are characterized by pathological protein aggregation leading to compromised proteostasis and eventual cell death [105]. The authors used *C. elegans* as a model organism to study how FIC-1-mediated AMPylation affects the dynamics of Amyloid-β (A*β*), polyglutamine repeat (poly-Q) and α-synuclein aggregation. Worm strains with constitutive muscular A*β* expression became paralysed and showed a significant reduction in lifespan, while Aβ-expressing strains additionally harbouring the constitutively active FIC-1^E274G^ showed increased lifespan and mobility relative to strains that expressed A*β* alone or were AMPylation-deficient [58]. Furthermore, RNAi mediated individual knockdown of HSP-1, HSP-3 and HSP-4 doubled survival, while simultaneous knock down of all three HSP70 family members increased worm survival approximately fourfold. Thioflavin S staining of amyloid plaques under inducing conditions revealed a significantly larger number of aggregates in worms expressing the hyperactive FIC-1 mutant as compared to other strains [58]. These observations imply that FIC-1 mediated AMPylation attenuates aggregation-induced toxicity through altering chaperone function, which in turn leads to the formation of larger cytoprotective aggregates as opposed to toxic oligomers.

Poly-Q diseases, such as Huntington's disease (HD), are the result of mutant, unstable poly-Q polypeptide aggregation [106]. Aggregation of PolyQ repeats is dependent on the age of animals and the length of such repeats [107]. While polyQ24YFP-expressing worms hardly show any aggregates till day 10 of adulthood, polyQ40-YFP-expressing animals already harbour discrete aggregates during larval development [108]. PolyQ40-YFP expressed in *fic-1* null animals showed an approximately twofold increase in the number of discrete loci on day 1 of adulthood as compared to WT worms expressing polyQ40-YFP. This difference became less conspicuous with age [58]. Further experiments to assess age-related changes in mobility and size of individual polyQ foci revealed that AMPylation-deficient animals contained a higher proportion of smaller aggregates on day 1. With age, however, the authors observed a higher proportion of larger aggregates (and reduced mobility) in these animals, similar to WT animals. Animals expressing the hyperactive FIC-1 mutant contained larger polyQ foci compared to AMPylation-deficient animals on day 9. However, these aggregates were significantly less mobile (by a factor of approx. 2) when compared with aggregates observed in AMPylation-deficient animals [58]. These observations suggest that AMPylation effects vary with the model being examined and the size of aggregates play an important role in determining animal fitness.

PD is characterized by the pathogenic aggregation of the presynaptic protein α-synuclein, which manifest as amyloid fibrils in Lewy bodies and Lewy neurites. These aggregates cause a progressive degeneration of dopaminergic neurons which leads to impaired motor functions and reflexes [109]. Expression of FIC-1(E274G) in α-synuclein-GFP-containing animals significantly expedited α-synuclein-GFP aggregate formation as compared to animals expressing only α-synuclein-GFP, while AMPylation-deficient animals had significantly lower number of aggregates as compared to controls [58]. As observed with the other models of NDs, larger aggregates proved beneficial for worm survival and motility. AMPylation-deficient animals, in this case, also showed enhanced survival but not to the extent seen for FIC-1 (E274G) animals [58]. Since AMPylation-deficient animals lack a putative negative regulator of chaperone function, and are not compromised in chaperone functions, *fic-1* knockout may enhance the cell's UPR systems and thus mitigate α-synuclein-GFP toxicity. Contrastingly, the expression of FIC-1(E274G) presumably leads to the reversible inhibition of certain HSP70 family chaperones (e.g. HSP-1, HSP-3, HSP-4) and possibly other proteins, which favours the formation of large, insoluble, α-synuclein-GFP aggregates that are less toxic or harmless for cells and could potentially be cleared through ubiquitin-mediated proteasomal degradation.

## 5. Conclusion and future perspectives

From bacterial toxin AMPylases that modulate host cytoskeletal architecture to metazoan AMPylases that regulate protein homeostasis in the ER, AMPylation plays a role in a diverse array of signalling pathways. The discovery of AMPylated proteins that are not likely to be modified by HYPE and a non-Fic AMPylase tempts us to speculate that there could be additional non-FicD AMPylases in human cells. In addition, many key questions regarding HYPE function remain to be addressed, such as the following. (a) Are there physiological cues and/or allosteric factors that mediate the switch between AMPylation/deAMPylation states in metazoan AMPylases? (b) Are there any dedicated de-AMPylases that mediate protein deAMPylation only? (c) Is HYPE capable of AMPylating additional ER-resident and/or non-ER targets? Answering these questions and more will increase our understanding of the impact of this modification on cellular physiology.

## Data accessibility

This article has no additional data.
